# A contribution to mayfly studies of Western Mongolia (Insecta, Ephemeroptera)

**DOI:** 10.3897/zookeys.638.10198

**Published:** 2016-12-08

**Authors:** Bolortsetseg Erdenee, Alain Maasri, Jon K. Gelhaus, Badamdorj Bayartogtokh

**Affiliations:** 1 Department of Biodiversity, Earth and Environmental Science, Drexel University, 3201 Arch Street, Suite 240, Philadelphia, PA 19104, USA; 2 Guest scientist at the Department of Ecosystem Research, Leibniz-Institute of Freshwater Ecology and Inland Fisheries (IGB), Müggelseedamm 301, DE-12587 Berlin, Germany; 3 Department of Entomology, Academy of Natural Sciences of Drexel University, 1900 Benjamin Franklin Parkway, Philadelphia, PA 19103, USA; 4 Department of Biology, School of Arts and Sciences, National University of Mongolia, Ulaanbaatar 210646, Mongolia

**Keywords:** Raptobaetopus tenellus, Caenis luctuosa, Caenis rivulorum, Biodiversity, Altai Mountain Range, Aquatic Insects

## Abstract

Streams in the Mongolian Altai Mountains are mostly fed from glaciers and are extreme conditions for mayflies because of high elevation, low temperatures and low annual precipitation. Previous information about mayflies of Western Mongolia is scarce, but with this study a total of 38 species belonging to 26 genera and subgenera and 8 families of mayflies has been recorded in the Mongolian Altai region. Study material was entirely imagos and collected from 78 sites during expeditions led by the Mongolian Aquatic Insect Survey in 2008, 2009 and 2010. *Raptobaetopus tenellus*, *Caenis luctuosa* and *Caenis rivulorum* are recorded as new to the fauna of Mongolia, and there are new distribution records for *Ameletus montanus*, Baetis (Acentrella) lapponica, *Baetis sibiricus*, Baetis (Labiobaetis) attrebatinus, *Centroptilum luteolum*, *Procloeon pennulatum*, *Ephemerella aurivillii*, *Serratella setigera*, *Ephemera sachalinensis*, Ecdyonurus (Afronurus) abracadabrus, *Cinygmula kurenzovi*, Ecdyonurus (Afghanurus) vicinus and Epeorus (Belovius) pellucidus from the Mongolian Altai region. *Baetis vernus* and *Ephemerella aurivillii* are the most frequently encountered species in this region.

## Introduction

Early studies of the mayfly fauna from the Mongolian region date to 1940 by Kinji Imanishi (Landa and Soldán 1983), although the major focus of this work consisted of exploring Inner Mongolia, a province of China, and to a lesser extent, the modern state of Mongolia (Bae et al. 2000). A decade later, Tshernova (1952) was the first to thoroughly describe the Mongolian (i.e., from the current Mongolian State) mayfly fauna with a paper including describing a new species, *Baetis mongolicus* (later, redescribed as a synonym of Baetis (Labiobaetis) tricolor by Kluge (2012)), from Khalkh gol, Eastern Mongolia. Most recently, Soldán et al. (2009), published a review of the Mongolian mayfly fauna and listed a total of 96 species belonging to 34 genera and 14 families. Of these, 28 species were recorded from Western Mongolia (defined as Uvs, Khovd and Bayan-Olgiy provinces of Mongolia).

Most of the area of Western Mongolia is highly elevated, mostly dominated by the Mongolian Altai Mountains, which have permanent glacial snow at the highest points. The average altitude of the Mongolian Altai Mountains is about 3200–3500 m a.s.l. The air temperature of the warmest month in Mongolian Altai Mountains is 12.3 °C in the higher areas and 21.1 °C in the lower areas of the region (Altantsetseg et al. 2008). Thus, for the region sampled for this study, we consider this area of high elevation and relatively cold summers as extreme conditions for mayflies.

Mayflies occur in variety of lotic and lentic environments and these habitats, including rivers, streams, springs and lakes, occur in Western Mongolia. The entire region of Western Mongolia is included within the Central Asian Internal Watershed (CAIW) (“Internal” from, Kelderman and Batima 2006; Maasri and Gelhaus 2012) which is one of the three major basins of Mongolia (Tsegmid 1969). The CAIW is an endorheic basin but equivalent to the size of the Arctic and Pacific Ocean basins of Mongolia (Dulmaa 1979). In this watershed, streams originating from glacial melt are common, in addition to lakes that originated from tectonic and glacial processes. The largest river by its discharge is Khovd gol (“gol” refers to stream or river in Mongolian) flowing for 516 km with a drainage area of 58000 km^2^ (Tsegmid 1969). The second largest river is Bulgan gol, which is 268 km long, and with a drainage area of about 9180 km^2^. The Bulgan gol originates from south of the Mongol Altai Mountains and flows west into the Urungu River of China. Bodonch gol and Uyench gol are the next largest rivers after Bulgan gol (Myagmarjav and Davaa 1999). Three out of the five largest lakes in Mongolia (as measured by surface area) occur in the CAIW specifically Uvs, Khyargas and Khar-Us lakes (the first two listed are salt water lakes, the last one is a freshwater lake). Uvs nuur (“nuur” refers to lake in Mongolian) is the largest lake in Mongolia, with a drainage basin of 70712 km^2^. In addition to these there are several smaller freshwater (Khoton nuur, Khorgon nuur, Dayan nuur and Achit nuur) and saltwater lakes (Uureg nuur) in the basin (Myagmarjav and Davaa 1999).

In this paper, we provide data on the species composition of mayflies in Western Mongolia and the Altai Mountains in order to contribute to the inventory of aquatic insect biodiversity in this relatively unexplored area of Mongolia and the larger Central Asian region. This study has the specificity to include a wide range of aquatic habitat types distributed along a wide latitudinal gradient.

## Materials and methods

### Study area

**Figure 1.**
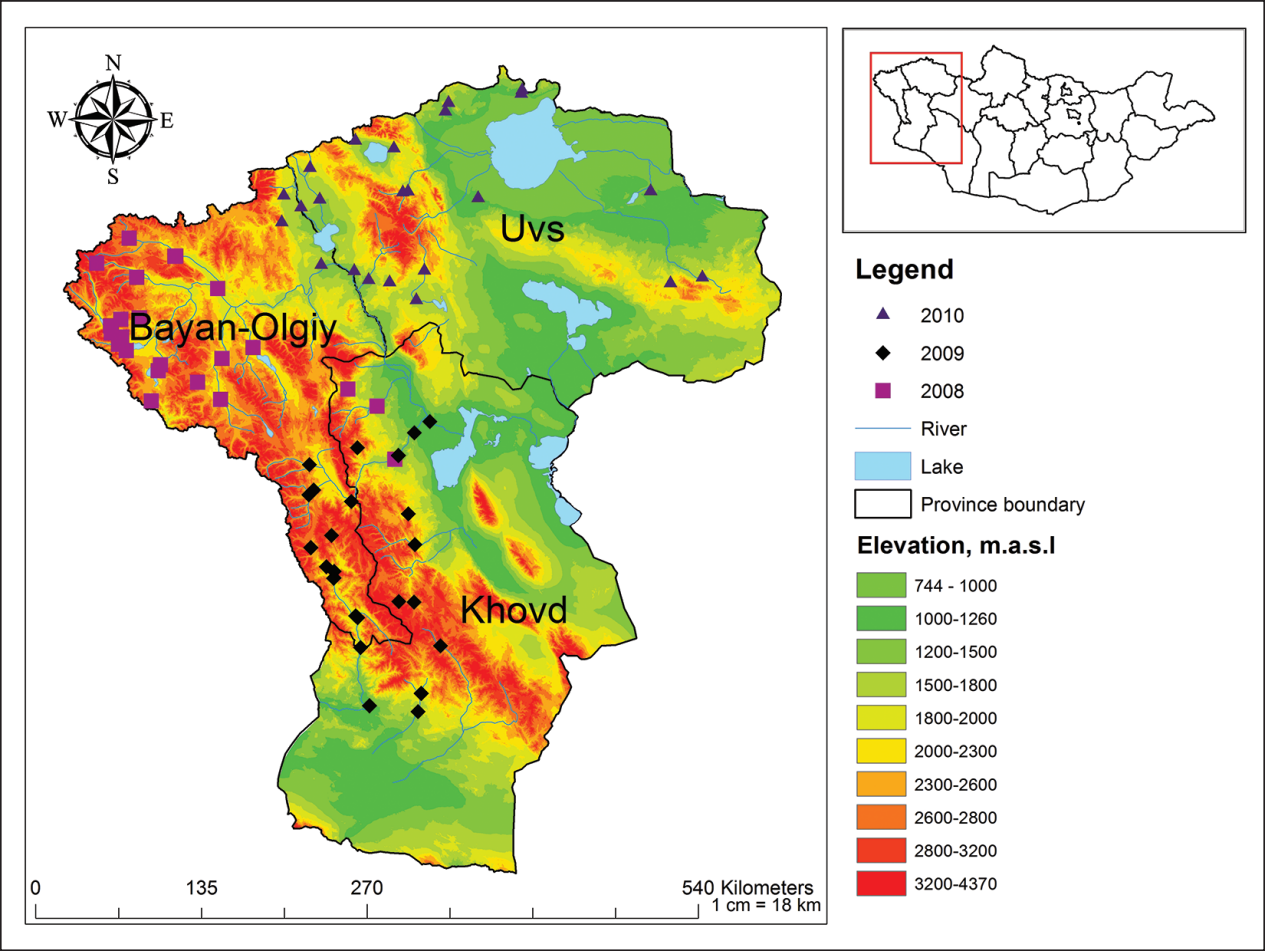
Sampling sites in the Mongolian Altai Mountain range (2008–2010).

**Figure 2.**
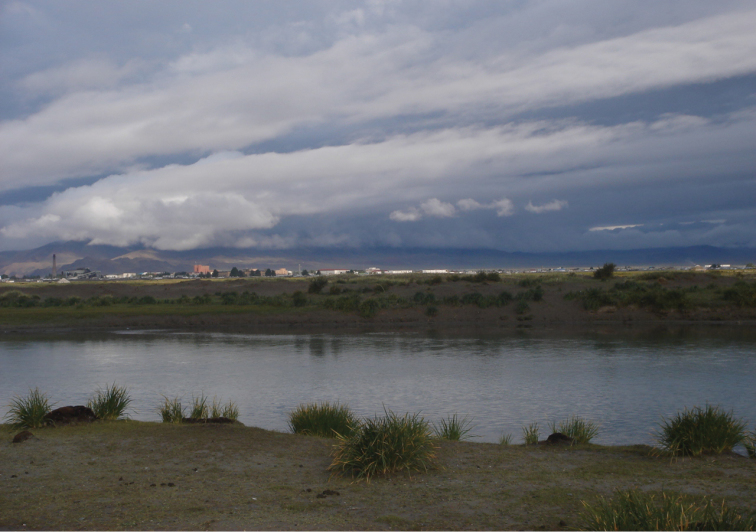
Khovd gol (site # 4).

**Figure 3.**
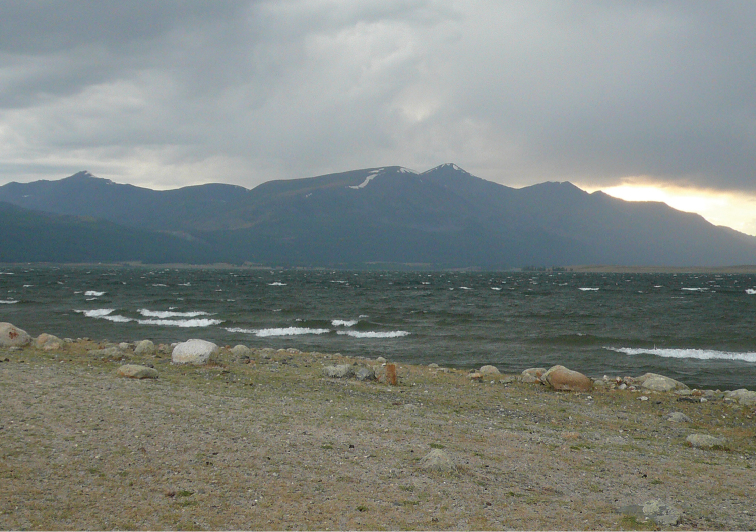
Khoton nuur (site # 13).

**Figure 4.**
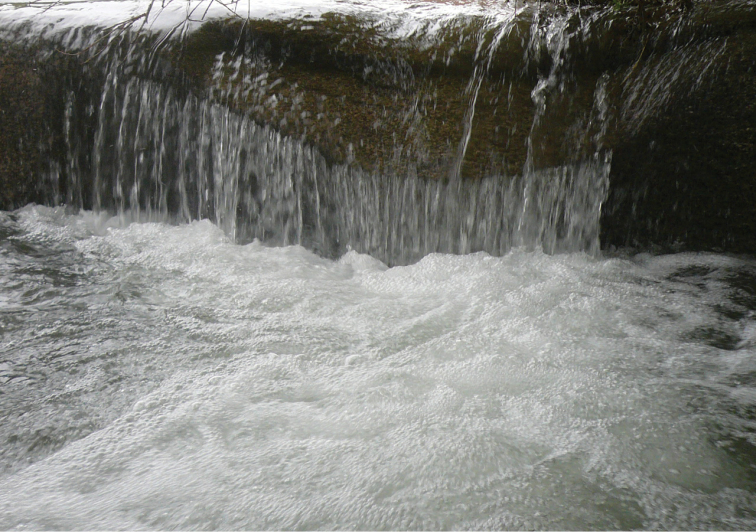
Uyench gol (site # 45).

**Figure 5.**
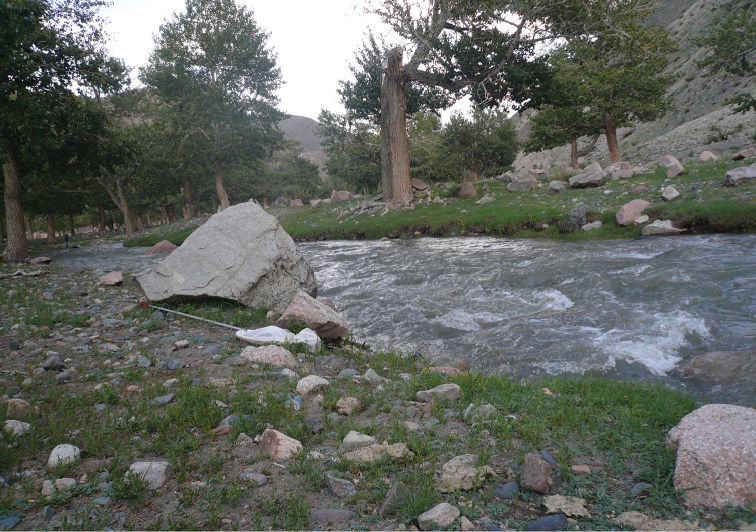
Bortiin gol (site # 49).

**Figure 6.**
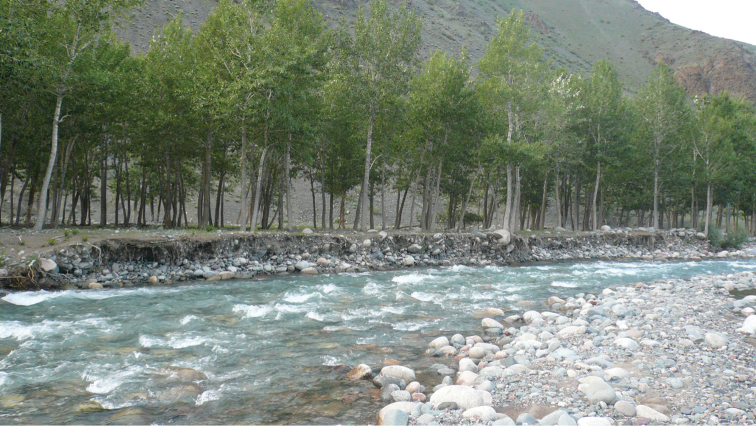
Turgen gol (site # 73).

**Figure 7.**
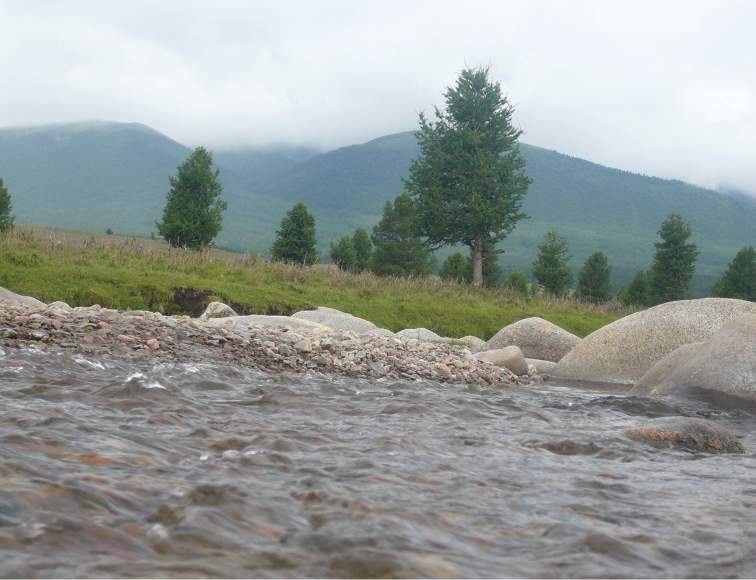
Baruunturuun gol (site # 77).

Mayfly samples were collected throughout the three provinces (aimags) of Western Mongolia, namely Khovd, Bayan-Olgiy and Uvs. We collected a total of 2180 adult specimens from 78 sites (Figure 1) in the Mongolian Altai mountain region, along streams, rivers, springs and several large lakes. Sampling sites included a wide range of elevation between 923 to 2798 m a.s.l, and a majority of streams and rivers (Figures 2 to 7, and Appendix 1).

### Sampling

Imago samples were collected in the framework of the Mongolian Aquatic Insect Survey (see Gelhaus 2012; Phillips-Iversion and Gelhaus 2010) in July of each year between 2008 and 2010. At each sampling site, sweep net and Malaise traps were used to collect mayfly imagos and occasionally white and black light traps were used to complement the collection. Two Malaise traps were set overnight directly along the stream channel with the head end of the trap adjacent to the stream bank. After collection, all specimens were preserved in the field in 80% ethanol solution. If subimagos were captured alive, they were kept in a dry place until the imago emerged.

Specimens were identified in the laboratory using a Leica EZ4 dissecting microscope and identification keys (Bajkova 1972; 1974; Kluge 1980; 1987; Tshernova 1952; 1964; Tshernova and Belov 1982). All specimens are preserved at the Institute of Meteorology, Hydrology and Environment, Ulaanbaatar, Mongolia.

## Results

**Figure 8.**
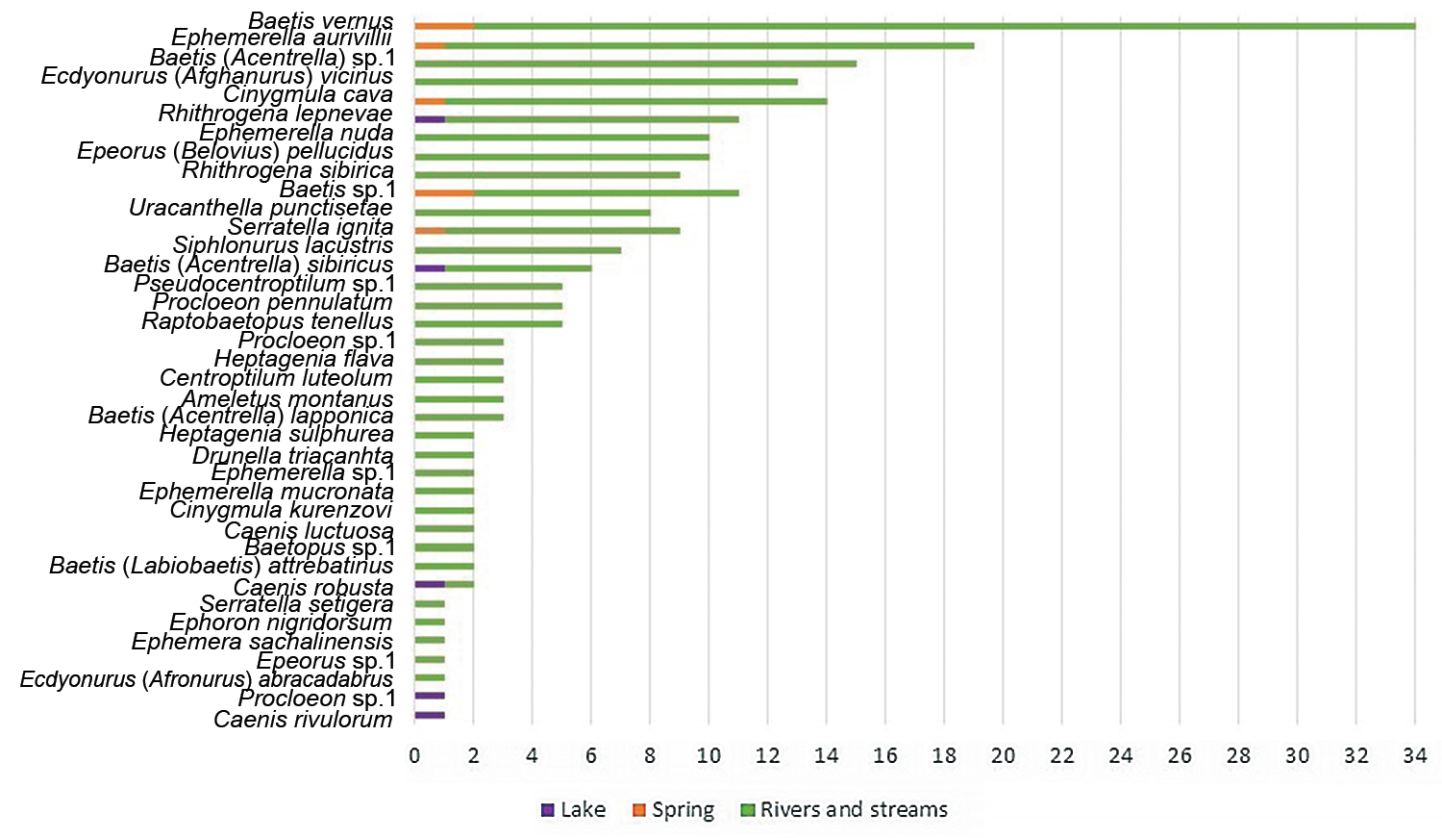
Species of mayflies recorded in Western Mongolia (ordered by the number of site occurrences). The different colors on the bar for each species represent the three main habitats and the length represents the number of occurrences for each type.

A total of 38 species, belonging to 26 genera and subgenera and 8 families of mayflies, are recorded in this study area (Figure 8). Among these, *Raptobaetopus tenellus* Albadra, 1878, *Caenis luctuosa* (Burmeister, 1839) and *Caenis rivulorum* Eaton, 1884 are new to the fauna of Mongolia, and there are new distribution records in Western Mongolia for 13 species: *Ameletus montanus* Imanishi, 1930, Baetis (Acentrella) lapponica Bengtsson, 1912, Baetis (Acentrella) sibiricus Kazlauskas, 1963, Baetis (Labiobaetis) attrebatinus Eaton, 1870, *Centroptilum luteolum* (Müller, 1776), *Procloeon pennulatum* (Eaton, 1870), *Ephemerella aurivillii* Bengtsson, 1909, *Serratella setigera* (Bajkova, 1965), *Ephemera sachalinensis* Matsumura, 1911, Ecdyonurus (Afronurus) abracadabrus (Kluge, 1983), *Cinygmula kurenzovi* (Bajkova, 1965), Ecdyonurus (Afghanurus) vicinus Demoulin, 1964, and Epeorus (Belovius) pellucidus (Brodsky, 1930). The following species list gives the specific localities where a species was found as site number (#), and Figure 8 ranks the species by number of sites where each species occurred. In the species list, preceding the species name, (*) refers to a new record for the Western Mongolia and (**) refers to a new record for the country.

**Ameletidae**

- * *Ameletus montanus* Imanishi, 1930 - # 22, 26, 31

**Baetidae**

- * Baetis (Acentrella) lapponica Bengtsson, 1912 - # 31, 51, 59

- Baetis (Acentrella) sp. 1 - # 1, 2, 4, 24, 27, 31, 33, 43, 47, 53, 56, 63, 65, 72, 77

- * Baetis (Acentrella) sibiricus Kazlauskas, 1963 - # 3, 16, 34, 35, 49, 50

- * Baetis (Labiobaetis) attrebatinus Eaton, 1870 - # 47, 70

- *Baetis vernus* Curtis, 1834 - # 2, 3, 7, 8, 9, 10, 12, 14, 15, 16, 18, 19, 20, 21, 22, 23, 24, 25, 29, 32, 35, 37, 38, 40, 45, 51, 52, 53, 56, 59, 61, 62, 63, 71

- *Baetis* sp. 1 - # 1, 3, 15, 18, 24, 41, 60, 62, 65, 66, 68

- *Baetopus* sp. 1 - # 60, 64

- * *Centroptilum luteolum* (Müller, 1776) - # 31, 37, 39

- * *Procloeon pennulatum* (Eaton, 1870) - # 59, 60, 61, 64, 66

- *Procloeon* sp. 1 - # 13

- *Pseudocentroptilum* sp. 1 - # 3, 22, 43, 48, 57

- *Pseudocloeon* sp. 1 - # 8, 16, 17

- ** *Raptobaetopus tenellus* Albadra, 1878 - # 57, 61, 63, 67, 71

**Caenidae**

- ** *Caenis luctuosa* (Burmeister, 1839) - # 16, 53

- ** *Caenis rivulorum* Eaton, 1884 - # 13

- *Caenis robusta* Eaton, 1884 - # 53, 58

**Ephemerellidae**

- *Drunella triacanhta* (Tshernova, 1949) - # 23, 41

- * *Ephemerella aurivillii* Bengtsson, 1909 - # 5, 6, 16, 19, 28, 31, 33, 35, 36, 37, 38, 41, 65, 67, 68, 70, 71, 73, 75

- *Ephemerella mucronata* (Bengtsson, 1909) - # 3, 38

- *Ephemerella nuda* Tshernova, 1949 - # 1, 3, 10, 11, 29, 30, 40, 53, 65, 66

- *Ephemerella* sp. 1 - # 30, 60

- *Serratella ignita* (Poda, 1761) - # 24, 43, 45, 54, 59, 61, 63, 66, 76

- * *Serratella setigera* (Bajkova, 1965) - # 43

- *Uracanthella punctisetae* Matsumura, 1931 - # 33, 37, 38, 39, 40, 41, 42, 43

**Ephemeridae**

- * *Ephemera sachalinensis* Matsumura, 1911 - # 43

**Heptageniidae**

- *Cinygmula cava* (Ulmer, 1927) - # 6, 17, 18, 19, 23, 31, 36, 65, 68, 69, 70, 73, 74, 75

- * *Cinygmula kurenzovi* (Bajkova, 1965) - # 4, 63

- * Ecdyonurus (Afghanurus) vicinus Demoulin, 1964 - # 18, 20, 22, 24, 45, 46, 47, 61, 65, 66, 72, 77, 78

- * Ecdyonurus (Afronurus) abracadabrus (Kluge, 1983) - # 43

- * Epeorus (Belovius) pellucidus (Brodsky, 1930) - # 3, 33, 36, 39, 40, 41, 42, 43, 47, 61

- *Epeorus* sp. 1 - # 19

- *Heptagenia flava* Rostock, 1878 - # 53, 54, 60

- *Heptagenia sulphurea* (Müller, 1776) - # 3, 53

- *Rhithrogena lepnevae* Brodsky, 1930 - # 2, 3, 24, 39, 40, 41, 42, 43, 44, 60, 78

- *Rhithrogena sibirica* Brodsky, 1930 - # 4, 19, 31, 32, 38, 55, 64, 67, 73

**Polymitarcyidae**

- *Ephoron nigridorsum* (Tshernova, 1934) - # 53

**Siphlonuridae**

- *Siphlonurus lacustris* Eaton, 1970 - # 12, 14, 56, 57, 71, 75, 78

Of the 38 species recorded as adults, 36 occurred along streams and rivers. Three species, *Caenis robusta*, Baetis (Acentrella) sibiricus and *Rhithrogena lepnevae*, were found along both lotic and lentic habitats. Only two species, *Procloeon* sp. and *Caenis rivulorum*, were recorded from a lake (# 13- Khoton nuur). Five species were taken around cold springs although none was found exclusively along this habitat.

The most frequently encountered species was *Baetis vernus*, which was recorded from 34 of the 78 sites (Figure 8). *Ephemerella aurivillii*, Baetis (Acentrella) sp. 1 and *Cinygmula cava* were found at 19, 15, and 14 different sites, respectively. In contrast, seven species were recorded as adults only at one site: *Serratella setigera* (site # 43), *Ephoron nigridorsum* (site # 53), *Ephemera sachalinensis* (site # 43), *Epeorus* sp. 1 (site # 19), Ecdyonurus (Afronurus) abracadabrus (site # 43), *Procloeon* sp. 1 (site # 13), and *Caenis rivulorum* (site # 13). The remaining species occurred at between two to 13 sites.

**Figure 9.**
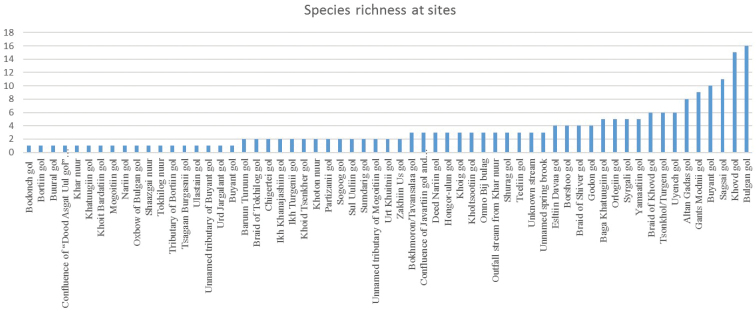
Number of species occurring at each site in Western Mongolia.

Taxa richness at rivers and lakes varied between one and 16 (Figure 9). The highest taxa richness was found along Bulgan gol (16 species) with 15 and 11 species along the rivers Khovd and Sagsai, respectively. The lowest species richness (one species) was observed at 18 rivers (e.g. Bodonch gol, Bortiin gol, Buural gol etc.).

## Discussion

Our study shows that the Ephemeroptera fauna of Western Mongolia comprise more than one-third of the total species recorded for the country. In addition, three new species were recorded for Mongolia for the first time, *Raptobaetopus tenellus*, *Caenis luctuosa* and *Caenis rivulorum*. *Raptobaetopus tenellus* is a Transpalaearctic (also referred as Entire Palaearctica, Beketov (2009)) species (Bauernfeind and Soldán 2012). Distribution of this species is known from west Palaearctic (Iberian Peninsula through Europe to the northern Ural Mountains) to Eastern Palaearctic (lower Ob' River and basin in Siberia to Primoriye region) (Bauernfeind and Soldán 2012). *Caenis luctuosa* and *Caenis rivulorum* are both Palaearctic species. *Caenis luctuosa* was known previously from Fennoscandia east to Russia and Middle Asia, south to the Balearic Islands, Iberian Peninsula and Asia minor, and some Mediterranean Islands and North Africa (Bauernfeind and Soldán 2012). *Caenis rivulorum* is a widespread species and is considered part of the Siberian fauna (Bauernfeind and Soldán 2012). *Ameletus montanus*, Baetis (Acentrella) lapponica, Baetis (Acentrella) sibiricus, Baetis (Labiobaetis) attrebatinus, *Centroptilum luteolum*, *Procloeon pennulatum*, *Ephemerella aurivillii*, *Serratella setigera*, *Ephemera sachalinensis*, Ecdyonurus (Afronurus) abracadabrus, *Cinygmula kurenzovi*, Ecdyonurus (Afghanurus) vicinus and Epeorus (Belovius) pellucidus are new to Western Mongolia. Of these, Baetis (Labiobaetis) attrebatinus, *Procloeon pennulatum*, *Ephemera sachalinensis*, Ecdyonurus (Afronurus) abracadabrus and Ecdyonurus (Afghanurus) vicinus were recently recorded in Mongolia for the first time by Enkhtaivan and Soldán (2004) and Soldán et al. (2009). The remaining species were previously known in Mongolia, from the Pacific Ocean basin and Arctic Ocean basin (Bajkova and Varykhanova 1978; Braasch 1986; Kluge 2009; Landa and Soldán 1983). Baetis (Acentrella) lapponica has been recorded previously in Mongolia (Kluge 2009) in the Selenge River Basin, based on imaginal, reared from larvae, records. However, this species was not included in the checklist of the mayflies of Mongolia (Soldán et al. 2009) due to incomplete locality records. Our finding of Baetis (Acentrella) lapponica in Western Mongolia, based on adult specimens confirms the species occurrence in Mongolia and brings the Mongolian mayfly fauna to 100 species.

Mayflies are generally diverse in lotic ecosystems as the majority of species prefer well-oxygenated habitat (Merritt et al. 2008). Consequently, the highest species diversities in this study were recorded along rivers, streams and springs. Fewer species including *Caenis robusta*, Baetis (Acentrella) sibiricus and *Rhithrogena lepnevae* were sampled around both lotic and lentic habitats. *Caenis robusta* was collected near a river (Khovd Gol) and also a brackish lake (Shaazgai nuur). Baetis (Acentrella) sibiricus and *Rhithrogena lepnevae* were found at more lotic habitats rather than lentic habitats. *Procloeon* sp. and *Caenis rivulorum* were recorded only in Lake Khoton. Larvae of *Caenis rivulorum* were previously recorded in lakes with stony substrate as well as rivers at variable elevations between 200–500 m a.s.l. in Europe (Bauernfeind and Soldán 2012). However, the elevation of Lake Khoton is 2086 m a.s.l. making this site the highest elevation record for the species.

*Baetis vernus* was the most commonly encountered taxon in Western Mongolia and occurred in 45% of the sampled sites. This species was found at a variety of lotic habitats including streams and springs. The elevation range of this species in Western Mongolia extended from 1172 to 2798 m a.s.l. The wide occurrence of this species among our sampled sites is most likely due to its very broad ecological range (Bauernfeind and Soldán 2012).

*Serratella setigera*, *Procloeon* sp. 1, *Ephoron nigridorsum*, *Ephemera sachalinensis*, *Epeorus* sp. 1, Ecdyonurus (Afronurus) abracadabrus, and *Caenis rivulorum* were found only at a single sampling site. *Ephoron nigridorsum* and *Ephemera sachalinensis* are both burrowing mayflies preferring larger and lowland rivers (Bauernfeind and Soldán 2012) and were recorded at Bulgan or Khovd River, the only suitable river habitat within the sampling area. For *Serratella setigera* and Ecdyonurus (Afronurus) abracadabrus, both found only at Bulgan Gol (Appendix 1, site # 43), this study adds significant habitat information to what little is known on the distribution of these two species (Bauernfeind and Soldán 2012).

## Conclusion

In this study a total of 38 species was recorded in Western Mongolia (Uvs, Khovd and Bayan-Olgiy provinces). Soldán et al. (2009) listed 28 species that have been recorded from the Khovd, Uvs and Bayan-Olgiy provinces, with more than half of these not collected in our sampling expeditions. Therefore, despite the valuable information taxonomic and geographical distribution of mayflies of Western Mongolia, this study does not constitute an inclusive checklist of the total mayfly fauna of Western Mongolia. This discrepancy could be related to a number of reasons due to sampling and current taxonomy. First, our sampling effort was restricted to July, a favorable period for emergence of aquatic insects in Mongolia, but nevertheless did not cover the complete ice-free period in Western Mongolia. Second, the sampling duration and number of samples at the different aquatic habitats was variable. Some rivers and streams were sampled thoroughly at different sites (e.g. Bulgan and Khovd River), others were only sampled overnight and in few sites was sampling occurring at the right timing during the day to encompass adult swarming. Therefore, our sampling might have been affected by different emergence patterns. Third, there were difficulties to identify some adult mayflies at the species level because of the lack of reliable identification keys for the Mongolia fauna and also having subimagos in the samples. Maasri and Gelhaus (2012) previously listed mayfly species based on larval identification and recorded 21 genera for the CAIW. However, Maasri and Gelhaus (2012) included sites throughout the whole CAIW, covering a wider geographical range. Erdenee (2011) in her previous study recorded 17 genera all included in this study. In addition to Soldán et al. (2009), Beketov (2005) in a survey of the Northeastern Altai Mountains recorded 25 species with 20 of these included in Western Mongolia. Therefore, our results and the available literature on Western Mongolia support the statement of an estimated number of mayfly species for this geographical area to be above 65 species.

## Appendix 1

**Table:** Study area and localities sampled for mayfly imagos in Western Mongolia. Soum refers to an administrative subunit of the aimag (or province). Dates are provided in day-month-year format.

Site number (#)	Site code in the MAIS database	GPS	Elevation, m.a.s.l	Site name	Province	Soum	Collection date	Habitat type
1	MAIS2008070301	48°19.26'N 91°18.53'E	1474	Shurag gol	Khovd	Erdenetburen	3.07.2008	Stream
2	MAIS2008070302	48°25.30'N 90°58.40'E	1805	Hongor-Ulun gol	Khovd	Erdenetburen	3–4.07.2008	Stream
3	MAIS2008070502	48°38.89'N 89°53.03'E	2065	Sagsai gol	Bayan-Olgiy	Sagsai	4–6.07.2008	River
4	MAIS2008070602	49°02.51'N 89°25.00'E	1775	Khovd gol	Bayan-Olgiy	Ulaankhus	6.07.2008	River
5	MAIS2008070701	49°14.28'N 88°54.43'E	2108	Sogoog gol	Bayan-Olgiy	Ulaankhus	7.07.2008	River
6	MAIS2008070702	49°14.09'N 88°54.01'E	2101	Sogoog gol	Bayan-Olgiy	Ulaankhus	7–8.07.2008	River
7	MAIS2008070802	49°19.11'N 88°21.98'E	2394	Ulastai gol	Bayan-Olgiy	Ulaankhus	8.07.2008	Stream
8	MAIS2008071001	49°06.27'N 88°02.91'E	2798	Sul Uuliin gol	Bayan-Olgiy	Tsengel	10.07.2008	Stream
9	MAIS2008071302	49°02.71N 88°30.53'E	2382	Khatuugiin gol	Bayan-Olgiy	Tsengel	13.07.2008	River
10	MAIS2008071304	48°45.53'N 88°36.06'E	2146	Unnamed tributary of Mogoitiin gol	Bayan-Olgiy	Tsengel	13–14.07.2008	Stream
11	MAIS2008071305	48°45.35'N 88°36.26'E	2142	Mogoitiin gol	Bayan-Olgiy	Tsengel	13–14.07.2008	Stream
12	MAIS2008071401	48°43.59'N 88°24.06'E	2431	Urt Khuitnii gol	Bayan-Olgiy	Tsengel	14.07.2008	River
13	MAIS2008071402	48°40.03'N 88°17.96'E	2086	Khoton nuur	Bayan-Olgiy	Tsengel	14–15.07.2008	Lake
14	MAIS2008071503	48°32.64'N 88°24.89'E	2147	Ikh Turgenii gol	Bayan-Olgiy	Tsengel	15–16.07.2008	Stream
15	MAIS2008071602	48°37.04'N 88°19.25'E	2115	Partizanii bulag	Bayan-Olgiy	Tsengel	16.07.2008	Spring
16	MAIS2008071603	48°35.92'N 88°26.21'E	2087	Syrgali gol	Bayan-Olgiy	Tsengel	16.07.2008	River
17	MAIS2008071604	48°30.38'N 88°30.57'E	2133	Sumdairag ol	Bayan-Olgiy	Tsengel	16–17.07.2008	Stream
18	MAIS2008071702	48°26.21'N 88°54.06'E	2232	Godon gol	Bayan-Olgiy	Sagsai	17.07.2008	River
19	MAIS2008071703	48°10.03'N 88°51.25'E	2065	Yamaatiin gol	Bayan-Olgiy	Sagsai	17–18.07.2008	Stream
20	MAIS2008071802	48°23.53'N 88°53.02'E	2184	Ikh Khanajashiin gol	Bayan-Olgiy	Sagsai	18.07.2008	Stream
21	MAIS2008071803	48°20.71'N 89°19.57'E	2422	Khoit Bardat gol	Bayan-Olgiy	Altai	18–19.07.2008	Stream
22	MAIS2008071901	48°14.47'N 89°36.10'E	2137	Sagsai gol	Bayan-Olgiy	Altai	19.07.2008	River
23	MAIS2008071902	48°32.48'N 89°33.60'E	2029	Kholtsootiin gol	Bayan-Olgiy	Buyant	19.07.2008	Stream
24	MAIS2008072002	47°56.87'N 91°33.48'E	1444	Buyant gol	Khovd	Khovd	20–21.07.2008	River
25	MAIS2009070101	47°58.59'N 91°35.48'E	1428	Buyant gol	Khovd	Khovd	01–03.07.2009	River
26	MAIS2009070201	48°00.19'N 91°08.46'E	2120	Unnamed tributary of Buyant gol	Khovd	Bayanbulag	2.07.2009	Stream
27	MAIS2009070403	47°34.87'N 91°10.23'E	2049	Buyant gol	Bayan-Olgiy	Deluun	03.07.2009	Stream
					Olgi			
28	MAIS2009070404	47°36.55'N 91°08.14'E	1947	Buyant gol	Bayan-Olgiy	Deluun	04.07.2009	River
29	MAIS2009070405	47°50.64'N 90°38.56'E	2165	Chigertei gol	Bayan-Olgiy	Deluun	04–05.07.2009	River
30	MAIS2009070501	47°37.47'N 90°40.32'E	2241	Gantsmodnii gol	Bayan-Olgiy	Deluun	05.07.2009	Stream
31	MAIS2009070502	47°39.84'N 90°43.10'E	2196	Gantsmodnii gol	Bayan-Olgiy	Deluun	05–06.07.2009	River
32	MAIS2009070602	47°20.80'N 90°57.61'E	2519	Confluence of “Dood Asgat Uul gol” and “Ulaagchiny Davaa gol” (two unnamed stream)	Bayan-Olgiy	Bulgan	06.07.2009	Stream
33	MAIS2009070604	47°05.32'N 91°01.61'E	2056	Bulgan gol	Bayan-Olgiy	Bulgan	06–07.07.2009	River
34	MAIS2009070703	47°14.48'N 90°45.19'E	2563	Khar nuur	Bayan-Olgiy	Bulgan	07.07.2009	Lake
35	MAIS2009070704	47°14.62'N 90°45.15'E	2560	Outfall stream from Khar nuur	Bayan-Olgiy	Bulgan	07.07.2009	Stream
36	MAIS2009070801	47°06.93'N 90°56.48'E	2122	Bulgan gol and roadside pools	Bayan-Olgiy	Bulgan	08.07.2009	Stream
37	MAIS2009070802	47°02.28'N 91°01.76'E	2016	“Elstiin Davaa” gol (unnamed stream)	Bayan-Olgiy	Bulgan	08–09.07.2009	Stream
38	MAIS2009070803	47°02.37'N 91°02.07'E	2010	Bulgan gol	Bayan-Olgiy	Bulgan	08–09.07.2009	River
39	MAIS2009070901	46°46.80'N 91°18.24'E	1801	Bulgan gol	Bayan-Olgiy	Bulgan	09.07.2009	River
40	MAIS2009070902	46°46.20'N 91°19.40'E	1792	Bulgan gol	Bayan-Olgiy	Bulgan	09–10.06.2009	River
41	MAIS2009070903	46°46.17'N 91°19.66'E	1788	Tsonkhol gol/Turgen gol (tributary of Bulgan gol)	Bayan-Olgiy	Bulgan	09–10.06.2009	Stream
42	MAIS2009071002	46°33.19'N 91°23.31'E	1509	Deed Nariin gol	Border of Bayan-Olgiy and Hovd	Bulgan	10.07.2009	Stream
43	MAIS2009071003	46°08.07'N 91°32.50'E	1200	Bulgan gol	Khovd	Bulgan	10–12.07.2009	River
44	MAIS2009071101	46°08.29'N 91°32.46'E	1210	Oxbow of Bulgan gol	Khovd	Bulgan	12.07.2009	Lake
45	MAIS2009071202	46°07.47'N 92°03.25'E	1470	Uyench gol	Khovd	Uyench	12–13-.07.2009	Stream
46	MAIS2009071301	46°15.63'N 92°04.37'E	1683	Urd Jargalant gol	Khovd	Uyench	13.07.2009	Stream
47	MAIS2009071302	46°15.66'N 92°04.36'E	1677	Uyench gol	Khovd	Uyench	13.07.2009	Stream
48	MAIS2009071401	46°37.21'N 92°13.84'E	2544	Bodonch gol	Khovd	Must	14.07.2009	Stream
49	MAIS2009071501	46°55.28'N 91°54.65'E	2311	Bortiin gol	Khovd	Munkhkhairkhan	15.07.2009	Stream
50	MAIS2009071503	46°54.89'N 91°44.83'E	2708	Tributary of Bortiin gol	Khovd	Munkhkhairkhan	15–16.07.2009	Stream
51	MAIS2009071701	47°20.40'N 91°51.79'E	1762	Khoid Tsenkher gol	Khovd	Duut	17–18.07.2009	Stream
52	MAIS2009071801	47°33.56'N 91°45.66'E	1865	Tsagaan Burgasnii gol	Khovd	Duut	18–19.07.2009	Spring
53	MAIS2009072002	48°14.74'N 91°54.09'E	1172	Khovd gol	Khovd	Khovd	20.07.2009	River
54	MAIS2009072003	48°09.20'N 91°44.58'E	1269	Buyant gol	Khovd	Khovd	20–21.07.2009	Stream
55	MAIS2009072101	47°58.56'N 91°35.80'E	1420	Buyant gol	Khovd	Buyant	21.07.2009	Stream
56	MAIS2010070402	49°20.40'N 91°40.90'E	1876	Orlogiin gol	Uvs	Umnugovi	04.07.2010	River
57	MAIS2010070403	49°07.10'N 91°37.51'E	1593	Orlogiin gol	Uvs	Umnugovi	04.07.2010	River
58	MAIS2010070502	49°13.70'N 91°18.64'E	1701	Shaazgai nuur	Uvs	Khovd	05.07.2010	Lake
59	MAIS2010070603	49°13.82'N 91°04.38'E	1475	Braid of Shiver gol	Uvs	Khovd	06.07.2010	Stream
60	MAIS2010070702	49°17.00'N 90°54.18'E	1405	Khovd gol bridge	Uvs	Khovd	07.07.2010	River
61	MAIS2010070802	49°18.12'N 90°31.78'E	1467	Braid of Khovd gol	Bayan-Olgiy	Nogoonnuur	08.07.2010	River
62	MAIS2010070803	49°34.66'N 90°02.10'E	1764	Zakhiin-Us gol	Bayan-Olgiy	Nogoonnuur	08–09.07.2010	Stream
63	MAIS2010070902	49°46.40'N 90°01.36'E	1694	Baga Khatuugiin gol	Bayan-Olgiy	Nogoonnuur	09–10.07.2010	River
64	MAIS2010071002	49°42.23'N 90°13.82'E	1526	Bokhmoron/Tavan salaa gol	Uvs	Bukhmurun soum	10.07.2010	River
65	MAIS2010071003	49°59.81'N 90°16.73'E	1763	Altan gadas gol	Uvs	Bukhmurun soum	10–11.07.2010	River
66	MAIS2010071101	49°46.55'N 90°25.87'E	1504	Altan gadas gol	Uvs	Bukhmurun soum	11.07.2010	River
67	MAIS2010071102	50°13.86'N 90°45.39'E	1552	Khoig gol	Uvs	Sagil	11.07.2010	River
68	MAIS2010071302	50°12.34'N 91°12.12'E	1637	Omno Bij bulag	Uvs	Sagil	13.07.2010	Spring
69	MAIS2010071402	50°43.04'N 92°35.96'E	1043	Tokhilog gol	Uvs	Davst	14.07.2010	River
70	MAIS2010071403	50°41.50'N 92°35.57'E	1003	Braid of Tokhilog gol	Uvs	Davst	14–15.07.2010	River
71	MAIS2010071502	50°34.57'N 91°46.22'E	1281	Borshoo gol	Uvs	Sagil	15–16.07.2010	Stream
72	MAIS2010071601	50°30.69'N 91°44.70'E	1229	Unnamed spring brook	Uvs	Sagil	17.07.2010	Stream
73	MAIS2010071602	49°53.54'N 91°21.14'E	1849	Confluence of Javartiin gol and Turgen gol	Uvs	Turgen	16–17.07.2010	River
74	MAIS2010071605	49°54.23'N 91°24.66'E	1812	Buural gol	Uvs	Turgen	17.07.2010	Stream
75	MAIS2010071701	49°54.24'N 92°12.51'E	955	Teeliin gol	Uvs	Tarialan	17–18.07.2010	River
76	MAIS2010071802	50°03.15'N 94°09.25'E	923	Nariin gol	Uvs	Zuungovi	18–19.07.2010	Spring
77	MAIS2010071902	49°26.76'N 94°47.76'E	1688	Baruun Turuun gol	Uvs	Undurkhangai	19–20.07.2010	River
78	MAIS2010072001	49°23.31'N 94°26.57'E	1832	Unknown stream	Uvs	Tsagaankhairkhan	20–21.07.2010	Stream
